# Artificial intelligence literacy and associations with thriving at work among nurses in Anhui Province, China: a latent profile analysis

**DOI:** 10.1186/s12912-026-05215-5

**Published:** 2026-08-10

**Authors:** Zhenni Xie, Jiangying Han, Dalin Kuang, Jinxiu Hu, Chenxia Liao, Ruiqi Wang

**Affiliations:** 1 Department of Nursing, The First Affiliated Hospital of Anhui Medical University, Hefei, Anhui 230001 China; 2 Department of General Thoracic Surgery, The First Affiliated Hospital of Anhui Medical University, Hefei, Anhui 230001 China

**Keywords:** Nurses, Artificial intelligence literacy, Thriving at work, Latent profile analysis, Associated factors

## Abstract

**Background:**

The integration of artificial intelligence (AI) technologies into clinical nursing is changing nursing practice and creating new competency requirements for nurses. Some nurses may still face difficulties in technical adaptation, ethical judgment, and practical use of AI tools in intelligent healthcare environments. Understanding nurses’ AI literacy and its relationship with thriving at work may help hospitals design more targeted support strategies.

**Aims:**

This study aimed to investigate the current status of nurses’ artificial intelligence literacy, identify latent profiles of self-reported AI literacy, analyze factors associated with profile membership, and examine differences in thriving at work across AI literacy profiles.

**Design:**

A cross-sectional study.

**Methods:**

In January 2026, 1000 nurses from 62 hospitals in Anhui Province, China were recruited by convenience sampling. Data were collected using a general information questionnaire, the Artificial Intelligence Literacy Scale, the Chinese version of the Thriving at Work Scale, a nine-item measure of attitudes toward AI in nursing, and the General Self-Efficacy Scale. Latent profile analysis was used to classify nurses’ AI literacy profiles. Factors associated with profile membership were examined using univariate analysis and multinomial logistic regression. Scores on the Thriving at Work Scale were compared across AI literacy profiles.

**Results:**

Three distinct latent profiles were identified: Ethical Awareness Deficit profile (*n* = 332, 33.20%), Cognition Practice Gap profile (*n* = 538, 53.80%), and Comprehensive Literacy Advantage profile (*n* = 130, 13.00%). Educational level, key nursing position/department management role, years of work experience, AI training experience, AI usage frequency in the past 6 months, attitude toward AI in nursing, and self-efficacy were associated with AI literacy profile membership (all *P* < 0.05). Scores on the Thriving at Work Scale differed significantly across the three profiles (*P* < 0.05).

**Conclusions:**

This multicenter cross-sectional study identified three latent profiles of self-reported artificial intelligence literacy among nurses from hospitals in Anhui Province, China. Thriving at work differed significantly across these profiles. The findings may inform stratified educational and managerial strategies to support nurses’ AI literacy and thriving at work in similar clinical contexts.

**Clinical trial number:**

Not applicable.

## Background

Artificial intelligence (AI) is increasingly being integrated into clinical nursing practice, including data analysis, risk warning, and decision support. These applications may improve nursing service efficiency and quality when used appropriately [1]. As AI becomes more embedded in nursing scenarios, nurses are expected not only to maintain traditional professional competencies but also to develop the ability to understand, evaluate, and use AI in intelligent healthcare environments. This competency is commonly referred to as AI literacy.

The term “AI literacy” was used by Agre [2] in an early technical guide for AI practitioners, although it referred mainly to familiarity with foundational AI literature rather than the broader competency-based concept used today. Ng et al. [3] defined AI literacy as the ability to understand and apply AI technologies without requiring prior technical expertise. Long et al. [4] further described it as a comprehensive capability that includes critical evaluation, effective collaboration, and appropriate application of AI technologies. In nursing, AI literacy can therefore be understood as nurses’ cognitive understanding, evaluative reflection, ethical judgment, and human-machine collaboration abilities required for delivering high-quality care in intelligent healthcare environments. Previous research suggests that higher AI literacy may help nurses use AI technologies more safely and effectively and support clinical decision-making and care quality [5]. However, current AI applications in nursing remain largely technology-driven, with relatively insufficient attention to nurses’ competency development. This may create a mismatch in which technological implementation advances faster than nurses’ AI-related preparedness. Nurses also differ in their awareness and understanding of AI technologies, and some may have difficulty recognizing how AI can support clinical work [6]. In addition, AI use in healthcare raises concerns about data privacy, algorithmic bias, and professional accountability, highlighting the need for nurses’ ethical awareness and judgment [7].

Thriving at work refers to a positive psychological state characterized by the joint experience of vitality and learning and is regarded as an important psychological resource for nurses’ professional development [8]. A recent three-wave longitudinal study found that AI literacy positively predicted career growth among new nurses through job crafting, indicating its potential relevance to nurses’ positive growth and development at work [9]. In AI-assisted healthcare environments, nurses with stronger AI literacy may be better prepared to engage with new technologies and may report greater professional confidence, creativity, and problem-solving capacity. Among nursing students, AI literacy has also been found to be closely related to academic adaptation and psychological resilience [10].

Existing research on nurses’ AI literacy remains at an early stage. Previous studies have relied on total scale scores, which may overlook within-group heterogeneity [5, 11]. Latent profile analysis (LPA) is a person-centered approach that identifies unobserved subgroups according to response patterns across multiple observed indicators. Compared with traditional grouping methods, LPA estimates profile membership probabilistically and uses model fit indices to guide the selection of the optimal profile solution [12]. This approach is therefore useful for identifying nurses with similar AI literacy patterns and for revealing heterogeneity that may be hidden behind similar total scores. Accordingly, this study used latent profile analysis to identify latent profiles of AI literacy among nurses in Anhui Province, China, and to compare thriving at work across profiles. The findings may provide empirical evidence for developing stratified educational, training, and managerial strategies to support nurses’ AI literacy development.

## Methods

### Subjects of investigation

In January 2026, convenience sampling was employed to recruit nurses from 62 hospitals in Anhui Province, China. Inclusion criteria were registered nurses working on-site for ≥ 6 months. Exclusion criteria included visiting or intern nurses. Sample size was calculated as 5–10 times the number of items [13]. With 52 questionnaire items and allowing for 20% invalid responses, the required sample was 325–650. In addition, according to Nylund’s recommendation [14], the minimum sample size for latent profile analysis should be 200 to ensure model stability. Ultimately, 1,026 questionnaires were collected, which met the sample size requirement.

### Survey instruments

#### General information questionnaire

The general information questionnaire captured participants’ sociodemographic, professional, institutional, and AI-related behavioral characteristics. It was developed through literature review and group discussion. Items included gender, age, marital status, educational level, professional title, hospital grade, hospital administrative affiliation, years of work experience, key nursing position/department management role, AI training experience, and AI usage frequency in the past 6 months.

### Artificial intelligence literacy scale

The Artificial Intelligence Literacy Scale (AILS), developed by Wang et al. [15] to assess artificial intelligence literacy in the general population, was used in this study. The scale comprises four dimensions: awareness, usage, evaluation, and ethics. The awareness dimension reflects an individual’s ability to recognize and understand AI-related technologies when encountering AI products; the usage dimension refers to the ability to efficiently complete tasks with the assistance of AI tools; the evaluation dimension reflects the ability to analyze, select, and critically judge AI applications and their outputs; and the ethics dimension focuses on users’ awareness of the potential risks and related responsibilities associated with AI technologies, as well as their adherence to relevant ethical principles. Each dimension contains three items, yielding a total of 12 items. The scale uses a 7-point Likert response format ranging from “strongly disagree” to “strongly agree,” scored from 1 to 7, with total scores ranging from 12 to 84; higher scores indicate higher levels of artificial intelligence literacy. The original scale showed good internal consistency, with a Cronbach’s alpha of 0.83. In the present study, the Cronbach’s alpha was 0.944. This scale was selected because its four dimensions correspond to foundational competencies required for the safe use of AI. However, as the AILS was not developed specifically for clinical nursing contexts, it may not fully capture more specific AI competencies required in nursing practice, such as integrating AI outputs into clinical decision-making, identifying patient safety-related risks, and adapting to AI-enabled nursing workflows. Therefore, in this study, AILS scores were interpreted as nurses’ self-reported general AI literacy rather than as an objective assessment of nursing-specific AI competence.

### Thriving at work scale

The Chinese version of the Thriving at Work Scale (TWS), originally developed by Porath et al. [16] and psychometrically evaluated among Chinese employees by Zeng et al. [17], was used to assess thriving at work. The scale comprises 10 items across two dimensions: learning and vitality, with five items in each dimension. Items 4 and 8 are reverse-scored. Each item is rated on a 7-point Likert scale ranging from 1 (strongly disagree) to 7 (strongly agree), yielding a total score of 10–70. Higher scores indicate greater thriving at work. Cronbach’s α was 0.827 in the present study.

### Attitudes toward AI in nursing

The nine-item section on attitudes toward AI in nursing from the questionnaire developed by Wang et al. [18] was used to assess nurses’ attitudes toward AI applications in nursing. Each item is rated on a 4-point Likert scale ranging from 1 (strongly disagree) to 4 (strongly agree). The total score ranges from 9 to 36, with higher scores indicating more positive attitudes toward AI applications in nursing. The original study reported a Cronbach’s α of 0.860, and Cronbach’s α was 0.921 in the present study.

### General self-efficacy scale

The General Self-Efficacy Scale (GSES), developed by Schwarzer et al. [19] and adapted into Chinese by Wang et al. [20], was used to assess nurses’ self-efficacy. The GSES is a unidimensional instrument consisting of 10 items, each scored on a 4-point Likert scale ranging from 1 (not at all true) to 4 (exactly true). Total scores range from 10 to 40, with higher scores indicating greater self-efficacy. The Chinese version demonstrated good internal consistency, with a Cronbach’s α of 0.87; Cronbach’s α was 0.813 in the present study.

### Data collection

Thirty nurses meeting the inclusion criteria participated in a pilot test before formal data collection. The pilot test was not intended to revalidate the standardized scales. Instead, because the General Information Questionnaire was developed for this study and included context-specific sociodemographic, occupational, AI training, and AI use items, the pilot test was used to assess the clarity and appropriateness of its wording and response options. It also assessed the comprehensibility of item instructions after scale integration, the estimated completion time, and the usability of the online Wenjuanxing (https://www.wjx.cn) survey process. Minor wording and layout adjustments were then made to improve readability. Participants in the pilot test were not included in the formal survey. Subsequently, with assistance from head nurses across departments, questionnaires were distributed and guidance was provided. The survey landing page explained the study purpose, questionnaire content, voluntary nature of participation, anonymity, confidentiality, and intended use of the data. It further clarified that the AI-related data collected in this study were limited to participants’ self-reported AI training experience, AI usage frequency in the past 6 months, AI literacy, and attitudes toward AI in nursing, rather than patient information, clinical records, or AI-system log data. Participants were informed that their responses would be used only for aggregate statistical analysis and academic research, and that no individual nurse or hospital would be identifiable in any report or publication. Participants proceeded to the questionnaire only after indicating informed consent online and could stop before submission. To ensure data integrity, all items were mandatory; incomplete submissions triggered automatic prompts preventing submission, and single-account restrictions prevented duplicate submissions. Two researchers monitored the data in real time and exported the dataset after one week with no new responses. After data collection, two researchers independently screened the submitted questionnaires for data quality by jointly considering completion time, response patterns, and logical consistency across related items. Disagreements were resolved through discussion until consensus was reached.

### Statistical analysis

Data were analyzed using SPSS 27.0 and Mplus 8.3. Common method bias was assessed using Harman’s single-factor test with unrotated exploratory factor analysis.

Latent profile analysis was conducted in Mplus 8.3 using the four AILS dimension scores as indicators to identify nurses’ AI literacy profiles. Models were estimated using robust maximum likelihood (MLR). One- to four-profile models were estimated and compared using the Akaike Information Criterion (AIC), Bayesian Information Criterion (BIC), sample-size adjusted BIC (aBIC), entropy, Lo-Mendell-Rubin likelihood ratio test (LMR-LRT), and bootstrapped likelihood ratio test (BLRT). Lower AIC, BIC, and aBIC values indicated better model fit; entropy values closer to 1 indicated higher classification precision; and significant LMR-LRT and BLRT results indicated that the k-profile model fitted better than the k-1 profile model. Because the four AILS dimensions were derived from the same scale, each contained three items, and all used the same 7-point response format, the raw dimension scores were considered comparable. In this study, measurement invariance testing was not conducted because LPA was used to identify latent subgroups within a single sample rather than to compare measurement structures across predefined groups. An indicator-scaling check and optimization-stability analyses were performed for the LPA. First, LPA was repeated using z-score standardized AILS dimension scores to examine whether the classification depended on indicator scaling. Second, each candidate model was estimated using 2,000 initial-stage random starts and 500 final-stage optimizations (STARTS = 2000 500), with 20 initial-stage iterations (STITERATIONS = 20) and a fixed random seed (STSEED = 20260712). Convergence and replication of the best log-likelihood were examined using TECH8. The selected three-profile model was then independently re-estimated using a different random seed (STSEED = 20260713).

Continuous variables were assessed for distributional characteristics before analysis. Normally distributed continuous variables were described as mean±standard deviation (SD), whereas non-normally distributed continuous variables were described as median [*P*25, *P*75]. Categorical variables were expressed as frequencies and percentages. For comparisons across latent profile groups, normally distributed continuous variables were compared using one-way analysis of variance, non-normally distributed continuous variables using Kruskal-Wallis H tests, and categorical variables using chi-square tests or Fisher’s exact tests, as appropriate. The distributional characteristics of the learning, vitality, and total scores on the Thriving at Work Scale were examined using skewness and kurtosis, and homogeneity of variance was assessed using Levene’s test. Because the homogeneity-of-variance assumption was violated for all three outcomes, differences in scores on the Thriving at Work Scale across the three AI literacy profiles were examined using Welch’s one-way analysis of variance, followed by Games-Howell post-hoc tests for pairwise comparisons. All tests were two-sided, and *P* < 0.05 was considered statistically significant.

For multinomial logistic regression, independent variables were selected using a comprehensive strategy that combined theoretical evidence with univariate results in parallel. Specifically, variables previously reported in the literature to be associated with artificial intelligence literacy [11, 21], together with variables showing statistically significant differences across latent profile groups in the univariate analysis (*P* < 0.05), were jointly included in the candidate variable pool. These two sources of evidence were considered simultaneously rather than through a stepwise screening procedure; that is, variables were not retained only after reaching statistical significance in univariate testing. Based on this strategy, age, educational level, work experience, key nursing position/department management role, AI training experience, AI usage frequency in the past 6 months, attitude toward AI in nursing, and self-efficacy were entered simultaneously into the multinomial logistic regression model using the enter method. Age, educational level, work experience, and AI usage frequency in the past 6 months were entered into the model as ordinal variables; their ORs therefore represent the change in the odds of profile membership associated with a one-category increase. Key nursing position/department management role and AI training experience were entered as binary categorical variables, with Yes as the reference category. Multicollinearity among the candidate predictors was assessed using tolerance and variance inflation factor (VIF) values. Serious multicollinearity was considered present when the tolerance value was < 0.10 and the VIF was > 10.

### Ethical approval

This study was conducted in accordance with the Declaration of Helsinki and was approved by the Ethics Committee of the First Affiliated Hospital of Anhui Medical University (approval no. KY2026-10-76). Data were collected anonymously through an online questionnaire. All participants provided online informed consent before completing the survey. All collected data were anonymized and used only for research purposes.

## Results

### Common method bias test

Harman’s single-factor test was conducted using unrotated exploratory factor analysis of the 41 items. The results showed that eight factors had eigenvalues greater than 1 and collectively explained 65.18% of the total variance. The first factor accounted for 19.91% of the variance, which was below the recommended threshold of 40% [22]. These findings indicate that no single dominant factor accounted for most of the covariance among the measured items, suggesting that serious common method bias was not evident in this study. Nevertheless, because all key variables were collected using self-reported questionnaires, the possibility of residual common method bias should still be considered when interpreting the findings.

### Characteristics of participants

After dual-review data-quality screening, 26 responses were excluded because of obvious random or patterned answering (*n* = 14), unusually short completion times combined with inadequate response quality (*n* = 7), or logical inconsistencies in core items (*n* = 5). Consequently, 1000 valid questionnaires were included in the analysis, yielding a valid response rate of 97.47%.

Participants were predominantly female (96.10%). The largest age group was nurses older than 30 and up to 40 years (52.00%), and most participants were married (79.90%). Educational level was relatively high, with 84.10% holding a bachelor’s degree or above. Regarding professional characteristics, the largest professional title category was nurse-in-charge (56.00%), and 52.40% of participants had more than 5 years of work experience. Most participants did not hold a key nursing position/department management role (72.70%). In terms of hospital characteristics, 58.20% of participants were from tertiary hospitals and 41.80% were from secondary hospitals; 45.50% were from provincial-level hospitals, 40.30% from prefecture-level hospitals, and 14.20% from county-level hospitals. Regarding AI training experience, 24.90% of participants reported having received AI training. For AI usage frequency in the past 6 months, weekly use was the most commonly reported category (39.50%), followed by monthly use (36.60%), occasional use (13.40%), and daily use (10.50%). Detailed participant characteristics are presented in Table 1.

**Table 1:** Participant characteristics (*N* = 1000)

Characteristic	*n* (%)
Gender	
Female	961 (96.10)
Male	39 (3.90)
Age (years)	
≤ 25	54 (5.40)
25 < age ≤ 30	252 (25.20)
30 < age ≤ 40	520 (52.00)
40 < age ≤ 50	130 (13.00)
> 50	44 (4.40)
Marital status	
Unmarried	201 (20.10)
Married	799 (79.90)
Educational level	
Junior college	159 (15.90)
Undergraduate	633 (63.30)
Master’s or above	208 (20.80)
Professional title	
Nurse	131 (13.10)
Senior nurse	193 (19.30)
Nurse-in-charge	560 (56.00)
Associate chief nurse	98 (9.80)
Chief nurse	18 (1.80)
Hospital grade	
Tertiary	582 (58.20)
Secondary	418 (41.80)
Hospital administrative affiliation	
County-level	142 (14.20)
Prefecture-level	403 (40.30)
Provincial-level	455 (45.50)
Work experience (years)	
≤ 1	117 (11.70)
1 < experience ≤ 3	145 (14.50)
3 < experience ≤ 5	214 (21.40)
5 < experience ≤ 10	274 (27.40)
> 10	250 (25.00)
Key nursing position/department management role	
No	727 (72.70)
Yes	273 (27.30)
AI training experience	
No	751 (75.10)
Yes	249 (24.90)
AI usage frequency in the past 6 months	
Occasionally	134 (13.40)
≥ 1/month	366 (36.60)
≥ 1/week	395 (39.50)
Daily	105 (10.50)

### AILS scores among nurses

The AILS total scores for 1000 nurses ranged from 42 to 80 (58.92 ± 9.28). Mean dimension scores were: awareness (14.59 ± 2.89), usage (14.93 ± 2.47), evaluation (15.09 ± 2.25), and ethics (14.32 ± 3.21).

### Latent profile analysis of nurses’ AI literacy

The primary LPA was conducted using the raw scores of the four AILS dimensions. Model fit indices for the one- to four-profile models are presented in Table 2. As the number of profiles increased, the AIC, BIC, and aBIC values decreased consistently, and the LMR-LRT and BLRT *P *values were < 0.001 for the two- to four-profile models, indicating better fit than the model with one fewer profile. However, model selection in LPA should not rely solely on information criteria, because these indices may continue to decrease as model complexity increases. Therefore, entropy, likelihood ratio tests, profile proportions, parsimony, classification certainty, and substantive interpretability were considered together.

The two-profile model had relatively higher AIC, BIC, and aBIC values and mainly provided a broad low-versus-high distinction, which was insufficient to capture heterogeneity in nurses’ AI literacy across the four dimensions. The three-profile model yielded the highest entropy (0.942), indicating the clearest classification among the candidate models. It also produced interpretable profile patterns, with acceptable profile proportions and practical relevance for nursing management. Although the four-profile model showed lower AIC, BIC, and aBIC values, its entropy declined to 0.884, suggesting reduced classification certainty. In addition, the four-profile solution increased model complexity but did not provide a clearer or more theoretically meaningful classification pattern. Considering model fit indices, entropy, likelihood ratio tests, profile proportions, parsimony, classification certainty, and substantive interpretability, the three-profile solution was selected as the optimal model.

After the three-profile solution was selected, additional checks were conducted to assess its stability. Each candidate model was estimated using 2,000 initial-stage random starts and 500 final-stage optimizations (STARTS = 2000 500; STITERATIONS = 20; STSEED = 20260712), and all models terminated normally. An independent run using a different random seed (STSEED = 20260713) reproduced the same log-likelihood, AIC (16693.322), BIC (16781.662), aBIC (16724.493), entropy (0.942), and profile sizes. After profile labels were matched according to the four AILS dimension means, all 1,000 participants had the same most likely profile assignment in the two runs. The four AILS dimension scores were then z-score standardized in Mplus 8.3, and LPA was repeated using the same random-start settings to examine whether the classification depended on indicator scaling. The standardized analysis also supported a three-profile solution. After profile labels were matched by the four AILS dimension means, agreement between the raw-score and standardized classifications was 100.00% (Cohen’s kappa = 1.000; adjusted Rand index = 1.000), and the profile sizes were identical: C1 = 332 (33.20%), C2 = 538 (53.80%), and C3 = 130 (13.00%). These results show that indicator rescaling did not change the classification in this dataset. Because z-score standardization is a linear transformation of the same indicators, this analysis was treated as a scaling check rather than independent evidence of model robustness.

**Table 2:** Fit indices for latent profile models of nurses’ AI literacy

Model	AIC	BIC	aBIC	Entropy	LMR-LRT (*P*)	BLRT (*P*)	Profile proportions (%)
1-class	19242.327	19281.589	19256.181	—	—	—	—
2-class	17524.553	17588.354	17547.065	0.914	< 0.001	< 0.001	36.72 / 63.28
3-class	16693.322	16781.662	16724.493	0.942	< 0.001	< 0.001	33.20 / 53.80 / 13.00
4-class	16440.132	16553.010	16479.961	0.884	< 0.001	< 0.001	27.99 / 25.56 / 33.54 / 12.91

### Characteristics and naming of nurses’ AI literacy profiles

**Fig. 1:**
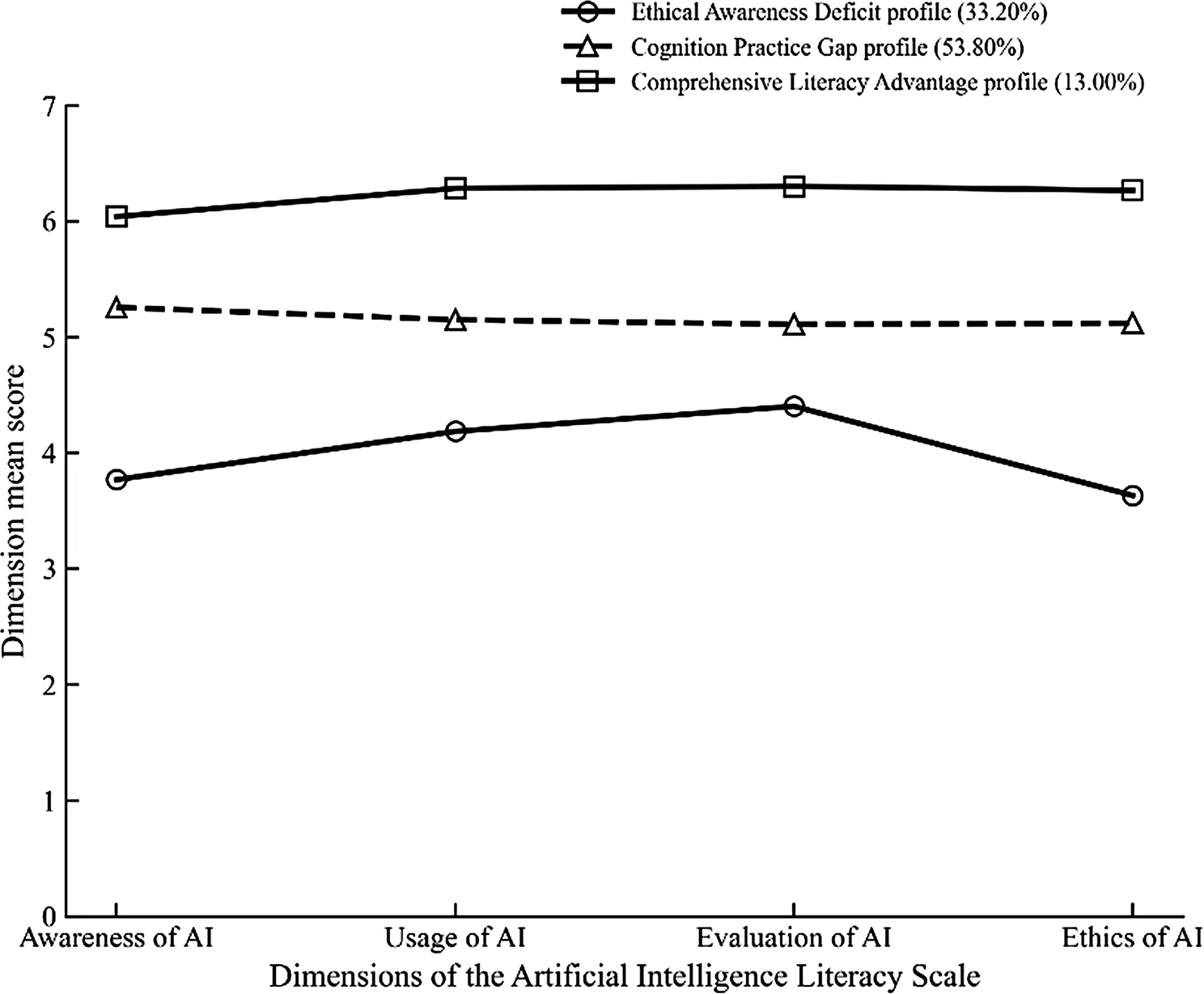
Latent profiles of artificial intelligence literacy among nurses in Anhui Province, China

Profile labels were assigned based on the relative score patterns across the four AILS dimensions, as shown in Fig. 1. For visualization, raw dimension scores were divided by three to obtain mean item scores; this linear transformation did not alter the relative profile patterns. Profile 1 included 332 nurses (33.20%) and showed the lowest overall scores, with a relatively lower ethics score; therefore, it was labeled the Ethical Awareness Deficit profile. Profile 2 included 538 nurses (53.80%) and showed intermediate overall scores, with relatively higher awareness but lower usage and evaluation scores, suggesting a gap between cognitive awareness and practical application; therefore, it was labeled the Cognition Practice Gap profile. Profile 3 included 130 nurses (13.00%) and showed consistently higher scores across all four dimensions, indicating a relatively balanced and advantageous literacy pattern; therefore, it was labeled the Comprehensive Literacy Advantage profile. These labels were used to summarize the observed patterns of self-reported AI literacy across the four AILS dimensions and should be understood as descriptive profile names.

### Thriving at work across AI literacy profiles

Scores on the Thriving at Work Scale differed significantly across the three AI literacy profiles. Welch’s one-way ANOVA showed significant between-profile differences in the learning score, Welch’s *F*(2, 436.225) = 17.425, *P* < 0.001; vitality score, Welch’s *F*(2, 480.272) = 26.726, *P* < 0.001; and total score, Welch’s *F*(2, 482.282) = 28.093, *P* < 0.001. Games-Howell post-hoc comparisons showed that nurses in the Comprehensive Literacy Advantage profile had significantly higher learning, vitality, and total scores on the Thriving at Work Scale than those in the Cognition Practice Gap and Ethical Awareness Deficit profiles. Nurses in the Cognition Practice Gap profile also scored significantly higher than those in the Ethical Awareness Deficit profile on all three outcomes (Table 3).

**Table 3:** Comparison of scores on the Thriving at Work Scale across nurses’ AI literacy profiles

Dimension	C1	C2	C3	Welch’s *F*	*P* value	*P*1 (C1 vs. C2)	*P*2 (C1 vs. C3)	*P*3 (C2 vs. C3)
Learning dimension	27.52 ± 3.70	28.49 ± 2.90	29.04 ± 1.80	17.425	< 0.001	< 0.001	< 0.001	0.020
Vitality dimension	26.60 ± 4.03	27.66 ± 3.52	28.57 ± 1.83	26.726	< 0.001	< 0.001	< 0.001	< 0.001
Total score	54.12 ± 7.13	56.16 ± 5.76	57.61 ± 2.98	28.093	< 0.001	< 0.001	< 0.001	< 0.001

Note: Data are presented as mean ± SD. Overall *P* values were obtained using Welch’s one-way ANOVA, and pairwise *P* values were obtained using Games-Howell post-hoc tests. C1 = Ethical Awareness Deficit profile; C2 = Cognition Practice Gap profile; C3 = Comprehensive Literacy Advantage profile

### Univariate analysis of factors associated with nurses’ AI literacy profiles

No significant differences were found across the three latent profiles in gender, age, marital status, professional title, hospital grade, or hospital administrative affiliation (all *P* > 0.05). In contrast, educational level, work experience, key nursing position/department management role, AI training experience, AI usage frequency in the past 6 months, attitude toward AI in nursing, and self-efficacy differed significantly across the three latent profiles (all *P* < 0.05). The full univariate comparisons are presented in Table 4.

**Table 4:** Univariate analysis of factors associated with nurses’ AI literacy profiles

Characteristic	C1 (*n* = 332)	C2 (*n* = 538)	C3 (*n* = 130)	Test statistic	*P*
Gender				*χ²*=5.29	0.071
Female	324 (97.59)	510 (94.80)	127 (97.69)		
Male	8 (2.41)	28 (5.20)	3 (2.31)		
Age (years)				*χ²*=4.30	0.829
≤ 25	18 (5.42)	29 (5.39)	7 (5.38)		
25 < age ≤ 30	96 (28.92)	126 (23.42)	30 (23.08)		
30 < age ≤ 40	161 (48.49)	289 (53.71)	70 (53.85)		
40 < age ≤ 50	43 (12.95)	71 (13.20)	16 (12.31)		
> 50	14 (4.22)	23 (4.28)	7 (5.38)		
Marital status				*χ²*=0.54	0.764
Unmarried	68 (20.48)	110 (20.45)	23 (17.69)		
Married	264 (79.52)	428 (79.55)	107 (82.31)		
Educational level				*χ²*=24.74	< 0.001
Junior college	68 (20.48)	83 (15.43)	8 (6.15)		
Undergraduate	209 (62.95)	345 (64.12)	79 (60.77)		
Master’s or above	55 (16.57)	110 (20.45)	43 (33.08)		
Professional title				*χ²*=13.72	0.089
Nurse	45 (13.55)	64 (11.90)	22 (16.92)		
Senior nurse	54 (16.27)	108 (20.07)	31 (23.85)		
Nurse-in-charge	198 (59.64)	304 (56.50)	58 (44.61)		
Associate chief nurse	32 (9.64)	52 (9.67)	14 (10.77)		
Chief nurse	3 (0.90)	10 (1.86)	5 (3.85)		
Hospital grade				*χ²*=1.20	0.548
Tertiary	194 (58.43)	318 (59.11)	70 (53.85)		
Secondary	138 (41.57)	220 (40.89)	60 (46.15)		
Hospital administrative affiliation				*χ²*=6.61	0.158
County-level	56 (16.87)	73 (13.57)	13 (10.00)		
Prefecture-level	139 (41.86)	215 (39.96)	49 (37.69)		
Provincial-level	137 (41.27)	250 (46.47)	68 (52.31)		
Work experience (years)				*χ²*=34.29	< 0.001
≤ 1	42 (12.65)	56 (10.41)	19 (14.62)		
1 < experience ≤ 3	41 (12.35)	76 (14.13)	28 (21.54)		
3 < experience ≤ 5	60 (18.07)	110 (20.45)	44 (33.84)		
5 < experience ≤ 10	96 (28.92)	154 (28.62)	24 (18.46)		
> 10	93 (28.01)	142 (26.39)	15 (11.54)		
Key nursing position/department management role				*χ²*=8.00	0.018
No	255 (76.81)	389 (72.30)	83 (63.85)		
Yes	77 (23.19)	149 (27.70)	47 (36.15)		
AI training experience				*χ²*=11.08	0.004
No	261 (78.61)	407 (75.65)	83 (63.85)		
Yes	71 (21.39)	131 (24.35)	47 (36.15)		
AI usage frequency in the past 6 months				*χ²*=27.87	< 0.001
Occasionally	49 (14.76)	70 (13.01)	15 (11.54)		
≥ 1/month	140 (42.16)	198 (36.80)	28 (21.54)		
≥ 1/week	121 (36.45)	211 (39.22)	63 (48.46)		
Daily	22 (6.63)	59 (10.97)	24 (18.46)		
Attitude toward AI in nursing score	24.58 ± 3.06	25.46 ± 3.22	26.24 ± 3.49	*F* = 14.71	< 0.001
Self-efficacy score	27.52 ± 3.36	28.30 ± 3.47	29.47 ± 3.35	*F* = 15.74	< 0.001

Note: C1 = Ethical Awareness Deficit profile; C2 = Cognition Practice Gap profile; C3 = Comprehensive Literacy Advantage profile

### Multicollinearity diagnosis of the independent variables

Before the multinomial logistic regression analysis, multicollinearity diagnostics showed that tolerance values ranged from 0.854 to 0.996 and VIF values ranged from 1.004 to 1.171. All tolerance values exceeded 0.10, and all VIF values were below 10, indicating no serious multicollinearity among the eight candidate predictors (Table 5).

**Table 5:** Multicollinearity diagnosis of the independent variables

Variable	Tolerance	VIF
Age (years)	0.941	1.063
Educational level	0.996	1.004
Work experience (years)	0.928	1.078
Key nursing position/department management role	0.854	1.171
AI training experience	0.866	1.155
AI usage frequency in the past 6 months	0.979	1.021
Attitude toward AI in nursing score	0.990	1.010
Self-efficacy score	0.980	1.020

### Multinomial logistic regression analysis of factors associated with nurses’ AI literacy profiles

Using AI literacy profile membership as the dependent variable, a multinomial logistic regression model was constructed. Independent variables were selected using a comprehensive strategy that combined theoretical evidence with univariate results in parallel. Specifically, variables previously reported in the literature to be associated with artificial intelligence literacy, together with variables showing statistically significant differences across latent profile groups in the univariate analysis (*P* < 0.05), were jointly included in the candidate variable pool. Based on this strategy, eight candidate variables were entered simultaneously into the model using the enter method: age, educational level, work experience, key nursing position/department management role, AI training experience, AI usage frequency in the past 6 months, attitude toward AI in nursing, and self-efficacy. The coding scheme for each variable is shown in Table 6. The results showed that age was not statistically significant, whereas the remaining seven variables showed statistically significant associations with nurses’ AI literacy profile membership in at least one profile comparison (Table 7).

**Table 6:** Coding scheme for independent variables

Variable	Coding
Age (years)	≤ 25 = 1; 25 < age ≤ 30 = 2; 30 < age ≤ 40 = 3; 40 < age ≤ 50 = 4; > 50 = 5
Educational level	Junior college = 1; Undergraduate = 2; Master’s or above = 3
Work experience (years)	≤ 1 = 1; 1 < experience ≤ 3 = 2; 3 < experience ≤ 5 = 3; 5 < experience ≤ 10 = 4; > 10 = 5
Key nursing position/department management role	No = 1; Yes = 2
AI training experience	No = 1; Yes = 2
AI usage frequency in the past 6 months	Occasionally = 1; ≥1/month = 2; ≥1/week = 3; Daily = 4
Attitude toward AI in nursing score	Original values
Self-efficacy score	Original values

**Table 7:** Multinomial logistic regression analysis of factors associated with nurses’ AI literacy profiles

Group	Variable	*β*	SE	Wald *χ*²	OR	95% CI	*P*
C1 versus C3¹	Constant	9.608	1.514	40.294	—	—	< 0.001
	Age (years)	-0.164	0.127	1.654	0.849	0.661–1.090	0.198
	Educational level	-0.884	0.191	21.334	0.413	0.284–0.601	< 0.001
	AI usage frequency in the past 6 months	-0.529	0.135	15.425	0.589	0.452–0.767	< 0.001
	Work experience (years)	0.387	0.086	20.041	1.473	1.243–1.745	< 0.001
	No key nursing position/department management role	1.223	0.280	19.123	3.399	1.964–5.881	< 0.001
	No AI training experience	1.104	0.278	15.732	3.017	1.748–5.208	< 0.001
	Attitude toward AI in nursing score	-0.156	0.035	19.550	0.856	0.799–0.917	< 0.001
	Self-efficacy score	-0.138	0.033	17.780	0.871	0.817–0.929	< 0.001
C2 versus C3¹	Constant	5.590	1.388	16.214	—	—	< 0.001
	Age (years)	-0.080	0.115	0.479	0.923	0.737–1.157	0.489
	Educational level	-0.625	0.176	12.553	0.535	0.379–0.756	< 0.001
	AI usage frequency in the past 6 months	-0.347	0.124	7.827	0.707	0.554–0.901	0.005
	Work experience (years)	0.355	0.079	20.392	1.426	1.222–1.663	< 0.001
	No key nursing position/department management role	0.936	0.258	13.158	2.549	1.537–4.226	< 0.001
	No AI training experience	0.874	0.256	11.630	2.397	1.450–3.962	< 0.001
	Attitude toward AI in nursing score	-0.071	0.032	4.857	0.932	0.875–0.992	0.028
	Self-efficacy score	-0.077	0.030	6.782	0.926	0.873–0.981	0.009
C1 versus C2²	Constant	4.018	0.950	17.869	—	—	< 0.001
	Age (years)	-0.084	0.086	0.946	0.919	0.776–1.089	0.331
	Educational level	-0.260	0.120	4.716	0.771	0.610–0.975	0.030
	AI usage frequency in the past 6 months	-0.182	0.085	4.559	0.834	0.705–0.985	0.033
	Work experience (years)	0.033	0.058	0.316	1.033	0.922–1.157	0.574
	No key nursing position/department management role	0.288	0.175	2.696	1.333	0.946–1.880	0.101
	No AI training experience	0.230	0.180	1.640	1.259	0.885–1.790	0.200
	Attitude toward AI in nursing score	-0.085	0.023	14.155	0.919	0.879–0.960	< 0.001
	Self-efficacy score	-0.060	0.021	8.051	0.942	0.903–0.982	0.005

Notes: C1 = Ethical Awareness Deficit profile; C2 = Cognition Practice Gap profile; C3 = Comprehensive Literacy Advantage profile. ^1^Reference = C3; ^2^Reference = C2; OR, odds ratio; SE, standard error. In the source data, No and Yes were coded as 1 and 2, respectively; in the regression analysis, Yes was specified as the reference category

## Discussion

### Nurses’ AI literacy showed heterogeneous profiles and requires targeted support

The mean total AILS score among the 1,000 nurses was 58.92 ± 9.28, indicating that their self-reported AI literacy was above the scale midpoint, a finding similar to that reported among pediatric nurses [23]. Latent profile analysis further showed clear heterogeneity in nurses’ self-reported AI literacy across the awareness, usage, evaluation, and ethics dimensions. The Ethical Awareness Deficit profile showed the lowest overall pattern, with a particularly low ethics dimension score. This pattern is consistent with previous concerns that nurses may need stronger preparation for patient data confidentiality, algorithmic bias, accountability, and patient safety in AI-assisted healthcare settings [24]. The Cognition Practice Gap profile showed basic awareness of AI but relatively lower usage and evaluation scores, suggesting that some nurses may have conceptual familiarity with AI but still face difficulties in applying AI tools or critically appraising AI outputs in clinical scenarios. In contrast, the Comprehensive Literacy Advantage profile showed higher scores across the awareness, usage, evaluation, and ethics dimensions, although it represented the smallest subgroup. These findings suggest that AI literacy support in nursing should not remain limited to general awareness. An integrated framework covering AI knowledge, practical skills, and ethical judgment may be considered. Educational innovation, clinical empowerment, and talent development could be coordinated to incorporate critical appraisal and ethical reasoning into routine training and practice.

### Differences in thriving at work across AI literacy profiles

In this study, nurses’ scores on the Thriving at Work Scale were in the moderate range, suggesting that further support for thriving at work may be needed. This finding is consistent with the results reported by Li et al. [25] among psychiatric nurses and by Terry et al. [26] among newly qualified nurses. Thriving at work is a positive psychological concept focused on work experience and derived from well-being psychology, incorporating both eudaimonic and hedonic perspectives of well-being [27]. In the present study, scores on the Thriving at Work Scale differed across the three AI literacy profiles, with higher scores observed in profiles characterized by stronger self-reported AI literacy. In intelligent nursing contexts, nurses with higher self-reported AI literacy may feel more able to adapt to AI-related tools, interpret AI-assisted information, and engage with technology-enabled workflows, which may be reflected in more favorable work experiences. This interpretation is consistent with Conservation of Resources theory [28], which emphasizes that individual resources are closely related to stress coping and positive psychological states. A study among undergraduate nursing students similarly reported associations between AI literacy, psychological empowerment, anxiety, and overall well-being [29]. Research involving university faculty has also found that higher AI literacy is associated with greater satisfaction with work autonomy, professional competence, interpersonal relationships, and work-life balance [30]. Future interventions could provide stratified AI literacy support according to profile characteristics. For nurses in the Ethical Awareness Deficit profile, hospitals could provide short 2- to 4-hour modules covering data privacy, algorithmic bias, patient safety, accountability, and basic AI operation, followed by case-based exercises using common clinical scenarios. For nurses in the Cognition Practice Gap profile, quarterly workshops or simulation-based training could focus on interpreting AI outputs, checking clinical relevance, identifying automation bias, and integrating AI suggestions with nursing judgment. For nurses in the Comprehensive Literacy Advantage profile, hospitals could encourage peer mentoring, ward-level demonstrations, and participation in AI-related quality-improvement projects. These measures may provide practical ideas for narrowing gaps in AI literacy across subgroups; however, whether such stratified support is associated with improvements in nurses’ thriving at work should be examined in future longitudinal or interventional studies.

### Factors associated with nurses’ AI literacy profiles

#### Sociodemographic and professional characteristics

Higher educational level was associated with a greater likelihood of belonging to the Cognition Practice Gap and Comprehensive Literacy Advantage profiles, which is consistent with the findings of Li et al. [31]. This association may be related to stronger knowledge reserves, more learning opportunities, and better information-processing abilities among nurses with higher educational level. It may also reflect greater exposure to emerging digital technologies and more opportunities to consider their integration into nursing work. In addition, nurses with shorter work experience were more likely to belong to the Comprehensive Literacy Advantage profile. Although longer work experience may indicate richer clinical experience, one possible explanation is that nurses with shorter tenure may be more receptive to emerging technologies. A study among midwives also showed that practitioners with 0–5 years of work experience held more positive attitudes toward AI [32]. Nurses without a key nursing position or department management role were more likely to belong to the Ethical Awareness Deficit profile. This association may reflect differences in opportunities to participate in AI training, quality-improvement activities, or decision-making processes, as well as differences in exposure to ethical and practical issues related to AI use. Similarly, Zeng et al. [33] reported higher AILS scores among teaching nurses, which may be related to routine training, instructional responsibilities, and continuing professional development opportunities. Nursing managers may therefore consider differentiated strategies, such as broadening continuing education opportunities for nurses with lower educational level, supporting senior nurses in updating digital mindsets, and providing standardized AI practical-skills training for general clinical nurses.

### AI training experience and usage frequency

Nurses without AI training were more likely to belong to the Ethical Awareness Deficit profile. This association may reflect fewer opportunities to learn basic AI concepts, algorithmic logic, clinical applicability, data-related risks, and ethical principles. A recent systematic review suggested that AI education and training may support the development of healthcare workers’ AI literacy by improving their attitudes and perceptions, AI-related knowledge and skills, and some behavioral outcomes [34]. The Technology Acceptance Model proposes that perceived usefulness and perceived ease of use are core factors related to technology adoption [35]. Specialized AI training may help strengthen nurses’ perceptions of technological usefulness, and hands-on training may support perceived ease of use and be related to the translation of use intentions into practical behavior. According to competency development theory, skill formation usually involves knowledge acquisition, internalization, and practice transfer. Differences in cognitive understanding and practical experience may therefore be reflected in group-level differences in AI literacy. However, most related studies have used cross-sectional designs, making it difficult to capture changes in literacy over time. Future longitudinal cohort studies, combined with structural equation modeling or latent growth modeling, could further examine how AI training, AI use, and AI literacy profiles are related over time.

Higher AI usage frequency was also associated with a greater likelihood of belonging to the Cognition Practice Gap and Comprehensive Literacy Advantage profiles. With the increasing implementation of big data and AI in healthcare, intelligent tools have received growing attention for their clinical reference value, and nurses may have more opportunities to encounter these tools in clinical work. In this study, 50.00% of nurses reported weekly or daily AI tool use. In a study of nursing students, Lan et al. [36] reported that frequent AI users had higher scores in AI cognition, data interpretation, and clinical application. However, without standardized guidance, frequent AI use may be accompanied by technology dependence or automation bias. Therefore, while promoting AI tools, clinical managers should also provide critical thinking training and AI ethics education to balance technology use with independent clinical judgment.

### Attitude toward AI in nursing and self-efficacy

Nurses with more positive attitudes toward AI in nursing were more likely to be classified into the Cognition Practice Gap and Comprehensive Literacy Advantage profiles. The Unified Theory of Acceptance and Use of Technology suggests that technology adoption is influenced not only by individual attitudes but also by performance expectancy and effort expectancy [37]. Nurses with positive attitudes toward AI in nursing may be more likely to recognize its value in clinical decision support and workflow optimization and to show a stronger willingness to learn about AI. Self-efficacy was also associated with profile membership: higher self-efficacy was associated with a greater likelihood of belonging to the Cognition Practice Gap and Comprehensive Literacy Advantage profiles, which is consistent with previous research among undergraduate nursing students [38]. Social Cognitive Theory states that self-efficacy is related to confidence in handling complex tasks and persistence in learning [39]. Nurses with stronger self-efficacy may report greater confidence and willingness to try when facing new AI-related tasks, as well as greater engagement in learning and practice. Previous studies have also reported an association between attitude toward AI and self-efficacy [40]. However, how attitudes toward AI in nursing, self-efficacy, and AI literacy profiles change together over time remains to be examined. Based on the associations observed in this study, AI literacy development should not be limited to single-skill training. Instead, an integrated cognitive, emotional, and behavioral intervention approach may be useful. Nursing managers could use clinical case demonstrations and real-world application examples to support nurses’ recognition of AI value, while providing tiered training and long-term support to reduce learning barriers and concerns about use. Such support may provide conditions for nurses to gradually build digital operation confidence and support the more standardized integration of AI into clinical nursing practice.

## Conclusions

This study identified three profiles of self-reported AI literacy among nurses in Anhui Province, China: the Ethical Awareness Deficit profile (33.20%), Cognition Practice Gap profile (53.80%), and Comprehensive Literacy Advantage profile (13.00%). Educational level, key nursing position/department management role, years of work experience, AI training experience, AI usage frequency in the past 6 months, attitude toward AI in nursing, and self-efficacy were associated with profile membership. Scores on the Thriving at Work Scale also differed significantly across the three profiles. These findings may inform differentiated educational and managerial strategies to support nurses’ AI literacy and thriving at work in similar clinical contexts.

### Limitations

Several limitations should be acknowledged. First, this study used convenience sampling and recruited nurses only from hospitals in Anhui Province, China. Although the sample included 1,000 nurses from 62 hospitals, selection bias may still exist, and the high proportion of female participants may limit generalizability. Anhui Province may also differ from other regions in per capita GDP, hospital digitalization, AI infrastructure, and nursing education resources. Therefore, the stability of the identified profiles and the transferability of the findings should be verified in multi-regional studies. Second, the profile labels were based on quantitative score patterns across the four AILS dimensions and should be interpreted as descriptive summaries of self-reported AI literacy rather than externally validated categories of nursing-specific AI competence. Self-reported AI literacy may not fully reflect actual AI-related ability and may be influenced by overestimation or social desirability. Although common method bias was assessed, residual bias cannot be completely excluded because all key variables were collected using self-report questionnaires. Future studies should incorporate objective indicators, such as AI knowledge tests, simulated clinical AI tasks, peer or supervisor assessments, and scenario-based evaluations. Third, the Comprehensive Literacy Advantage profile included 130 nurses, accounting for 13.00% of the sample. Parameter estimates involving this smaller subgroup may be less stable, especially when multiple predictors are examined simultaneously. Findings related to this profile should therefore be interpreted cautiously and verified in larger independent samples. Fourth, this study did not include organizational-level variables, such as hospital AI infrastructure, institutional support, department culture, leadership support, and the degree of AI integration into clinical workflows. Future studies should collect hospital- and department-level data and use multilevel models to examine how organizational context is related to nurses’ AI literacy profiles and work-related outcomes.

## Data Availability

The data supporting this study’s findings are available from the corresponding author upon reasonable request. Due to participant privacy protection and data confidentiality requirements, the data are not publicly available.

## References

[1] Loutfy A, Elzeiny A, Foster M, Alkubati SA, Sarman A, Tuncay S, et al. Artificial intelligence in pediatric nursing and its education: a systematic review. J Pediatr Nurs. 2026;88:542–50. 10.1016/j.pedn.2026.03.027. 41894996

[2] Agre PE. What to read: a biased guide to AI literacy for the beginner. Cambridge (MA): MIT Artificial Intelligence Laboratory; 1982. AI Working Paper No. 239. https://hdl.handle.net/1721.1/41185.

[3] Ng DTK, Leung JKL, Chu SKW, Qiao MS. Conceptualizing AI literacy: an exploratory review. Computers Education: Artif Intell. 2021;2:100041. 10.1016/j.caeai.2021.100041.

[4] Long D, Magerko B. What is AI literacy? Competencies and design considerations. In: proceedings of the 2020 CHI conference on human factors in computing systems. Honolulu, HI, USA: ACM; 2020;1–16. 10.1145/3313831.3376727.

[5] Özçevik Subaşi D, Akça Sümengen A, Semerci R, Şimşek E, Çakır GN, Temizsoy E. Paediatric nurses’ perspectives on artificial intelligence applications: A cross-sectional study of concerns, literacy levels and attitudes. J Adv Nurs. 2025;81(3):1353–63. 10.1111/jan.16335. 39003632

[6] El Arab RA, Alshakihs AH, Alabdulwahab SH, Almubarak YS, Alkhalifah SS, Abdrbo A, et al. Artificial intelligence in nursing: a systematic review of attitudes, literacy, readiness, and adoption intentions among nursing students and practicing nurses. Front Digit Health. 2025;7:1666005. 10.3389/fdgth.2025.1666005. PMC12507812 41079691

[7] El-Sayed AAI, Alsenany SA, Badoman TEA, Asal MGR. Development and Validation of a Scale for Nurses’ Ethical Awareness in The Use of Artificial Intelligence: A Methodological Study. Nurs Health Sci. 2025;27(2):e70140. 10.1111/nhs.70140. 40414799

[8] Zeng H, Zhao L, Zhao Y. Inclusive Leadership and Taking-Charge Behavior: Roles of Psychological Safety and Thriving at Work. Front Psychol. 2020;11:62. 10.3389/fpsyg.2020.00062. PMC7044413 32153448

[9] Liu D, Li W, Lu G, Ma Z, Jing J, Qiu H, et al. Dynamic changes and relationships among AI Literacy, job crafting, and career growth in new nurses in the AI era: A multicenter three-wave longitudinal study. Nurse Educ Today. 2026;167:107237. 10.1016/j.nedt.2026.107237. 42341711

[10] Chen Y, Lv Z, Li X, Chen Y, Li W. Artificial intelligence literacy and academic resilience in undergraduate nursing students: the mediating role of self-efficacy and artificial intelligence anxiety. BMC Nurs. 2026;25(1):276. 10.1186/s12912-026-04420-6. PMC13020335 41715113

[11] Kahraman H, Akutay S, Yüceler Kaçmaz H, Taşci S. Artificial Intelligence Literacy Levels of Perioperative Nurses: The Case of Türkiye. Nurs Health Sci. 2025;27(1):e70059. 10.1111/nhs.70059. PMC11825190 39947206

[12] Mori M, Krumholz HM, Allore HG. Using Latent Class Analysis to Identify Hidden Clinical Phenotypes. JAMA. 2020;324(7):700–1. 10.1001/jama.2020.2278. 32808993

[13] Ni P, Chen JL, Liu N. The sample size estimation in quantitative nursing research. Chin J Nurs. 2010;45(4):378–80. (in Chinese).

[14] Nylund KL, Asparouhov T, Muthén BO. Deciding on the number of classes in latent class analysis and growth mixture modeling: A Monte Carlo simulation study. Struct Equ Model. 2007;14(4):535–69.

[15] Wang B, Rau PLP, Yuan T. Measuring user competence in using artificial intelligence: validity and reliability of artificial intelligence literacy scale. Behav Inf Technol. 2023;42(9):1324–37.

[16] Porath C, Spreitzer G, Gibson C, Garnett FG. Thriving at work: Toward its measurement, construct validation, and theoretical refinement. J Organ Behav. 2012;33(2):250–75.

[17] Zeng LP, Wang YY, Zeng DP, She A, Lan W. Validity and reliability of the Chinese version of the Thriving at Work Scale among Chinese employees. Chin J Clin Psychol. 2020;28(4):730–33, 704. (in Chinese).

[18] Wang X, Fei F, Wei J, Huang M, Xiang F, Tu J, et al. Knowledge and attitudes toward artificial intelligence in nursing among various categories of professionals in China: a cross-sectional study. Front Public Health. 2024;12:1433252. 10.3389/fpubh.2024.1433252. PMC11250283 39015390

[19] Schwarzer R, Born A. Optimistic self-beliefs: Assessment of general perceived self-efficacy in thirteen cultures. World Psychol. 1997;3(1):177–90.

[20] Wang CK, Hu ZF, Liu Y. Evidences for reliability and validity of the Chinese version of the General Self-Efficacy Scale. Chin J Appl Psychol. 2001;7(1):37–40. (in Chinese).

[21] Liu S, Xiao Y, Nie M, Yuan X, Wang L, Wang M, et al. Nurses’ attitudes toward artificial intelligence: AI literacy as a predictor and the mediating effect of AI anxiety. BMC Nurs. 2025;24(1):1511. 10.1186/s12912-025-04142-1. PMC12752257 41272773

[22] Zhou H, Long LR. Statistical remedies for common method biases. Adv Psychol Sci. 2004;12(6):942–50. (in Chinese).

[23] Uludaşdemir D, Ayar G, Yetkin EE. The relationship between artificial intelligence literacy and artificial intelligence anxiety: A cross-sectional study among pediatric nurses. J Pediatr Nurs. 2026;88:664–70. 10.1016/j.pedn.2026.03.037. 41935428

[24] Abuhammad S. Strengthening ethical practices of patient data confidentiality and sharing among nurses in the artificial intelligence-driven healthcare era. SAGE Open Nurs. 2025;11:23779608251398113. 10.1177/23779608251398113. PMC12623646 41262122

[25] Li J, Li X, Chen Y, Chen M, Shi W, Huang H. The mediating role of thriving at work between emotional labor and presenteeism: a comparison between psychiatric nurses with and without sleep disturbances. BMC Psychiatry. 2025;26(1):106. 10.1186/s12888-025-07744-w. PMC12859898 41466395

[26] Terry D, Jacobs S, Moloney W, East L, Ryan L, Elliott J, et al. The Impact of Thriving at Work and Occupational Supports: Early Career Nurse Intentions to Leave an Organisation and Profession. J Adv Nurs. 2025;81(12):8608–19. 10.1111/jan.16817. PMC12623713 39930315

[27] Bai B, Qiao J, Bai C. Harnessing strengths from trauma: examining the impact of strength use on nurses’ job satisfaction, positive mental health, and thriving at work through post-traumatic growth. BMC Nurs. 2025;24(1):438. 10.1186/s12912-025-02936-x. PMC12007320 40251504

[28] Hobfoll SE. Conservation of resources. A new attempt at conceptualizing stress. Am Psychol. 1989;44(3):513–24. 10.1037//0003-066x.44.3.513 2648906

[29] Alshowkan A, Shdaifat E. Impact of AI Literacy on Well-Being Among Nursing Students-Mediating Roles of Empowerment and Anxiety: Cross-Sectional Study. JMIR Nurs. 2025;8:e79789. 10.2196/79789. PMC12721487 41428941

[30] Huang L, Zhao Y. The impact of AI literacy on work-life balance and job satisfaction among university faculty: a self-determination theory perspective. Front Psychol. 2025;16:1669247. 10.3389/fpsyg.2025.1669247. PMC12487956 41040088

[31] Li J, Liu Y, Xiang L, Liang G. Artificial intelligence literacy and influencing factors among clinical nurses in southeastern China: a latent profile analysis. BMC Nurs. 2026;25(1):716. 10.1186/s12912-026-04872-w. PMC13474754 42271407

[32] Unlu Bidik N, Turan Z. Examining the relationship between midwives’ attitudes toward artificial intelligence use and their job satisfaction. BMC Nurs. 2025;24(1):771. 10.1186/s12912-025-03346-9. PMC12210978 40597300

[33] Zeng Q, Huang X, Zhu J, Su S, Hu Y, Zhang X. Mechanisms of nurses’ AI use intention formation in Sichuan, Yunnan, and Beijing, China: mediating effects of AI literacy via self-efficacy-to-attitude pathways. Front Public Health. 2025;13:1622802. 10.3389/fpubh.2025.1622802. PMC12287104 40709030

[34] Woods L, Lyons K, Van Der Vegt A, Olsen Q, Huang W, Khor JS, et al. Assessing the effectiveness of artificial intelligence education and training for healthcare workers: a systematic review. BMC Med Educ. 2026;26(1):549. 10.1186/s12909-026-08969-3. PMC13045066 41807961

[35] Kumpulainen H, Kinnunen UM, Jylhä V. ICT acceptance in social and healthcare context - A scoping review of theories and models. Int J Med Inf. 2025;195:105796. 10.1016/j.ijmedinf.2025.105796. 39826407

[36] Lan X, Li M, Wang Y, Chen M, Jiang H, Lu S, et al. Characteristics and determinants of artificial intelligence (AI) literacy in Chinese nursing students: A cross-sectional study. Int J Nurs Stud Adv. 2026;10:100482. 10.1016/j.ijnsa.2026.100482. PMC12828738 41584432

[37] Venkatesh V, Morris MG, Davis GB, Davis FD. User acceptance of information technology: toward a unified view. MIS Q. 2003;27(3):425–78. 10.2307/30036540.

[38] Zhu S, Li R, Ren X, Huo J, Tan Q, Hu X, et al. A latent profile analysis of artificial intelligence literacy among undergraduate nursing students: a cross-sectional study. BMC Nurs. 2026;25(1):156. 10.1186/s12912-026-04331-6. PMC12905945 41559744

[39] Bandura A. Self-efficacy: toward a unifying theory of behavioral change. Psychol Rev. 1977;84(2):191–215. 10.1037//0033-295x.84.2.191 847061

[40] Gu Y, Xiong B, Li R, Pan Z, Huang Y, Pan Y. Understanding secondary school students’ intentions to learn artificial intelligence: a multigroup structural equation modeling analysis. Front Psychol. 2026;17:1833613. 10.3389/fpsyg.2026.1833613. PMC13337926 42416165

